# Genetic variants in Hippo pathway genes are associated with house dust mite‐induced allergic rhinitis in a Chinese population

**DOI:** 10.1002/clt2.12077

**Published:** 2021-12-28

**Authors:** Min Chen, Rui Zheng, Fei Li, Jun‐Yi Xin, Si‐Lu Chen, Xin‐Jie Zhu, Xiang Gu, Meng‐Di Dai, Yi‐Fan Yang, Hai‐Yan Chu, Zheng‐Dong Zhang, Mei‐Ping Lu, Lei Cheng

**Affiliations:** ^1^ Department of Otorhinolaryngology & Clinical Allergy Center The First Affiliated Hospital, Nanjing Medical University Nanjing China; ^2^ Department of Environmental Genomics Jiangsu Key Laboratory of Cancer Biomarkers, Prevention and Treatment, Collaborative Innovation Center for Cancer Personalized Medicine, Nanjing Medical University Nanjing China; ^3^ Department of Genetic Toxicology The Key Laboratory of Modern Toxicology of Ministry of Education, Center for Global Health, School of Public Health, Nanjing Medical University Nanjing China; ^4^ Department of Otorhinolaryngology The Affiliated YiLi Friendship Hospital, Nanjing Medical University Yining China; ^5^ International Centre for Allergy Research, Nanjing Medical University Nanjing China

**Keywords:** allergic rhinitis, *DLG5*, genetic variation, Hippo pathway, mites, risk, Acariens, *DLG5*, La voie de signalisation Hippo, Rhinite allergique, Risque, Variation génétique

## Abstract

**Background:**

House dust mite (HDM)‐induced allergic rhinitis (AR) is a highly prevalent disease with bothersome symptoms. Genetic variants of the Hippo pathway genes play a critical role in the respiratory disease. However, no study has reported associations between variants of the Hippo pathway genes and HDM‐induced AR risk.

**Methods:**

Forty‐three key genes in the Hippo pathway were selected from the Kyoto Encyclopedia of Genes and Genomes (KEGG), Reactome pathway database, and previous reported studies. A case‐control study of 222 cases and 237 controls was performed to assess the associations between 121 genetic variants in these genes and HDM‐induced AR risk. DNeasy Blood & Tissues Kits were used for extracting genomic DNA from the venous blood and Infinium Asian Screening Array BeadChips for performing genotyping. A logistic regression model was applied to evaluate the effects of variants on HDM‐induced AR risk. The false discovery rate (FDR) method was utilized to correct for multiple testing. The receiver operating characteristic (ROC) curve was plotted to obtain the cut‐off value of total IgE for the diagnosis of HDM‐induced AR. Histone modification and transcription factor binding sites were visualized by UCSC genome browser. Moreover, expression qualitative trait loci (eQTL) analysis was obtained from Genotype‐Tissue Expression (GTEx) database.

**Results:**

We found that rs754466 in *DLG5* was significantly associated with a decreased HDM‐induced AR risk after FDR correction (adjusted odds ratio [OR] = 0.52, 95% confidence interval [CI] = 0.36–0.74, *p* = 3.25 × 10^−4^, *P*_FDR_ = 3.93 × 10^−2^). The rs754466 A allele reduced the risk of HDM‐induced AR in the subgroup of moderate/severe total nasal symptom score (TNSS). Furthermore, rs754466 was associated with a high mRNA expression of *DLG5*. Additionally, histone modification and transcription factor binding sites were rich in the region containing rs754466.

**Conclusion:**

Our findings indicated that rs754466 in *DLG5* decreased the susceptibility to HDM‐induced AR.

## 1 INTRODUCTION

Allergic rhinitis (AR) is often triggered after exposure to indoor or outdoor aeroallergens, such as house dust mite (HDM), pollens, animal dander and fungal allergens.^1^ HDM is almost omnipresent and perennial indoors, leaving no effective measures to eliminate HDM. Additionally, constant exposure to HDM, compared with other aeroallergens, may contribute to more chronic and severe symptoms that exert long‐term impact on quality of life.^2^, ^3^, ^4^ Moreover, HDM‐induced AR can increase the risk of asthma.^5^ Many studies have verified the critical role of genetic variants in the pathogenesis of AR.^6^ This inspired us to delve into HDM‐induced AR mechanism through genetic variations.

The Hippo pathway, first identified in *Drosophila melanogaster*, is an evolutionarily conserved pathway regulating organ size, tissue generation, and stem cell self‐renewal.^7^, ^8^ Dysregulation of the Hippo pathway is widely implicated in cancers,^9^ cardiac diseases,^10^ renal diseases,^11^ and respiratory diseases.^12^, ^13^, ^14^ Previous studies suggested that single‐nucleotide polymorphisms (SNPs) in *YAP*, *FRMD6*, *BIRC5*, and *DLG2* of the Hippo pathway were associated with asthma risk.^13^, ^15^, ^16^ To date, no study has been undertaken to investigate the associations between genetic variants in the Hippo pathway and HDM‐induced AR risk.

In this study, we hypothesized that genetic variants in the Hippo pathway were associated with HDM‐induced AR risk, and tested this hypothesis with a case‐control study among Han Chinese.

## 2 MATERIALS AND METHODS

### 2.1 Study population

A total of 222 cases and 237 controls were included in this case‐control study. The cases were recruited from the First Affiliated Hospital of Nanjing Medical University between May 2008 and October 2017. The diagnosis of HDM‐induced AR was established according to Allergic Rhinitis and its Impact on Asthma criteria.^1^ All cases were allergic simultaneously to *Dermatophagoides pteronyssinus* (*Der p*) and *Dermatophagoides farinae (Der f)*. Cases with asthma, atopic dermatitis, sinusitis, other nasal disorders, and malignant diseases were excluded. The healthy controls were randomly selected from physical examination centers in the same geographical region from April 2015 to October 2019 and with matched age (±5 years) and sex. The healthy controls met all the following criteria: (1) no symptoms of nasal disorders; (2) no symptoms and family history of atopic diseases; (3) specific allergen‐IgE < 0.35 kU_A_/L measured by the Phadiatop test (ThermoFisher Scientific, Uppsala, Sweden). All the individuals were Han Chinese and signed informed consent. Approximately 5 ml of peripheral venous blood was donated by each individual. The research approval was obtained from the ethics committee at Nanjing Medical University.

### 2.2 Clinical evaluation

A total nasal symptom score (TNSS) on a scale of 0–12 evaluating sneezing, rhinorrhea, nasal itching and nasal obstruction was used to assess the disease severity. The score of each symptom is: 0 = no symptoms; 1 = mild symptoms (symptom clearly present, but easily tolerated); 2 = moderate symptoms (definite awareness of symptom, i.e., bothersome but tolerable); 3 = severe symptoms (symptom, i.e., hard to tolerate; causes interference with activities of daily living and/or sleeping).^17^ Cases with TNSS of 0–4 and 5–12 were diagnosed with mild and moderate/severe HDM‐induced AR, respectively.^18^ The serum total IgE was measured by ImmunoCAP system (ThermoFisher Scientific, Uppsala, Sweden). Serum specific IgEs to *Der p*, *Der f*, cat dander, dog dander, *Blatella germanica*, *Alternaria alternate*, *Ambrosia elatior*, and *Artemisia vulgaris* were also detected in cases by ImmunoCAP system. Phadiatop tests were conducted in controls.

### 2.3 Selection of genes and genetic variants in the Hippo pathway

Forty‐three key genes in the Hippo pathway were selected from the Kyoto Encyclopedia of Genes and Genomes (KEGG), Reactome Pathway database, and reported studies.^15^, ^16^, ^19^, ^20^, ^21^, ^22^, ^23^ Genes located on sex chromosomes were excluded. Next, the data of Han Chinese in Beijing (CHB) and Japanese in Tokyo (JPT) from the 1000 Genomes Project (March 2012) was used to identify genetic variants. Then, variants matching the following quality control criteria were selected: minor allele frequency (MAF) > 0.05, call rate > 95%, and *p* value of Hardy–Weinberg equilibrium (HWE) > 10^−6^. After that, pairwise linkage disequilibrium (LD) analysis (*r* ^*2*^ ≥ 0.8) was performed to obtain tagging SNPs by Haploview 4.2 software. Finally, web‐based tools (SNPinfo Web Server (https://snpinfo.niehs.nih.gov/), HaploReg (http://pubs.broadinstitute.org/mammals/haploreg/haploreg.php), and RegulomeDB (https://www.regulomedb.org/regulome‐search/)) were used to predict putative functions of genetic variants. In addition, genetic variants with RegulomeDB Score ≥6 were removed.

### 2.4 SNP genotyping

Genomic DNA was successfully extracted from the venous blood of all individuals using DNeasy Blood & Tissue Kits (QIAGEN, Hilden, Germany). Genotyping was performed by Infinium Asian Screening Array BeadChips (Illumina, Inc., San Diego, CA, United States). The genetic variants and samples were selected according to a uniformed quality control protocol.

### 2.5 In silico analysis

Histone modification and transcription factor binding sites were visualized by UCSC genome browser from chromatin immunoprecipitation sequencing (ChIP‐Seq) data stored in ENCODE. The expression qualitative trait loci (eQTL) analysis was obtained from Genotype‐Tissue Expression (GTEx) database (https://www.gtexportal.org/home/) for genetic variants.

### 2.6 Statistical analysis

Student's *t‐*test was used for continuous variables and Chi‐square test for categorical variables to compare the demographic distribution and clinical variables between cases and controls. HWE in control group was calculated by a goodness‐of‐fit *χ* ^*2*^ test. The receiver operating characteristic (ROC) curve was plotted to obtain the cut‐off value of total IgE for the diagnosis of HDM‐induced AR. The levels of total IgE and specific IgEs to both *Der p* and *Der f* were analyzed after logarithmic transformation. The logistic regression model was performed to calculate adjusted and crude odds ratios (ORs) and their 95% confidence intervals (CIs). The false discovery rate (FDR) method was utilized to restrict the probability of false‐positive findings, due to the large number of genetic variants selected. The heterogeneity was tested with Cochran Q‐test and *I* ^*2*^ statistic. *p* < 0.05 was considered as a statistically significant level. All statistical analyses were computed by R 3.6.2 and PLINK v1.09.

## 3 RESULTS

### 3.1 Characteristics of study population

The demographic and clinical characteristics of 222 cases and 237 healthy controls are summarized in Table 1. No significant differences in age (*p* = 0.739) and sex (*p* = 0.974) were found between cases and controls. The level of total IgE was significantly higher in cases than in controls (*p* < 0.001). According to the ROC curve (Figure S1), the cut‐off value of total IgE was set as 60.45 kU/L, with <60.45 kU/L in 44 cases and ≥60.45 kU/L in 178 cases. The mean values of specific IgE to *Der p* and specific IgE to *Der f* were 1.26 and 0.97, respectively. Among 222 cases, 180 were diagnosed with moderate/severe HDM‐induced AR.

**TABLE 1:** Characteristics of HDM‐induced AR cases and controls

Variables	Cases (%), *n* = 222	Controls (%), *n* = 237	*p*
Age, years (mean ± SD)	23.23 ± 12.18	23.61 ± 12.40	0.739
Sex			0.974
Male	128 (57.7)	137 (57.8)	
Female	94 (42.3)	100 (42.2)	
Total IgE^a^ (log kU/L, mean ± SD)	2.09 ± 0.47	1.33 ± 0.63	< 0.001
Low^b^	44 (19.8)		
High^c^	178 (80.2)		
Specific IgE^a^ (log kU_A_/L, mean ± SD)			
*Der p*	1.26 ± 0.59		
Grade 1–3	102 (45.9)		
Grade 4–6	120 (54.1)		
*Der f*	0.97 ± 0.63		
Grade 1–3	147 (66.2)		
Grade 4–6	75 (33.8)		
TNSS			
Mild^d^	42 (18.9)		
Moderate/severe^e^	180 (81.1)		
Symptom: sneezing			
Mild	59 (26.6)		
Moderate/severe	150 (67.6)		
None	13 (5.8)		
Symptom: rhinorrhea			
Mild	52 (23.4)		
Moderate/severe	160 (72.1)		
None	10 (4.5)		
Symptom: nasal itching			
Mild	62 (27.9)		
Moderate/severe	127 (57.2)		
None	33 (14.9)		
Symptom: nasal obstruction			
Mild	57 (25.7)		
Moderate/severe	137 (61.7)		
None	28 (12.6)		

Abbreviations: AR, allergic rhinitis; *Der f*, *Dermatophagoides farinae*; *Der p*, *Dermatophagoides pteronyssinus*; HDM, house dust mite; SD, standard deviation; TNSS, total nasal symptom score.

^a^ The levels of total IgE and specific IgEs were log transformed to normalize the distribution.

^b^ Total IgE < 60.45 kU/L.

^c^ Total IgE ≥ 60.45 kU/L.

^d^ TNSS ≤ 4.

^e^ TNSS > 4.

### 3.2 Genetic variant in *DLG5* and HDM‐induced AR risk

**FIGURE 1:**
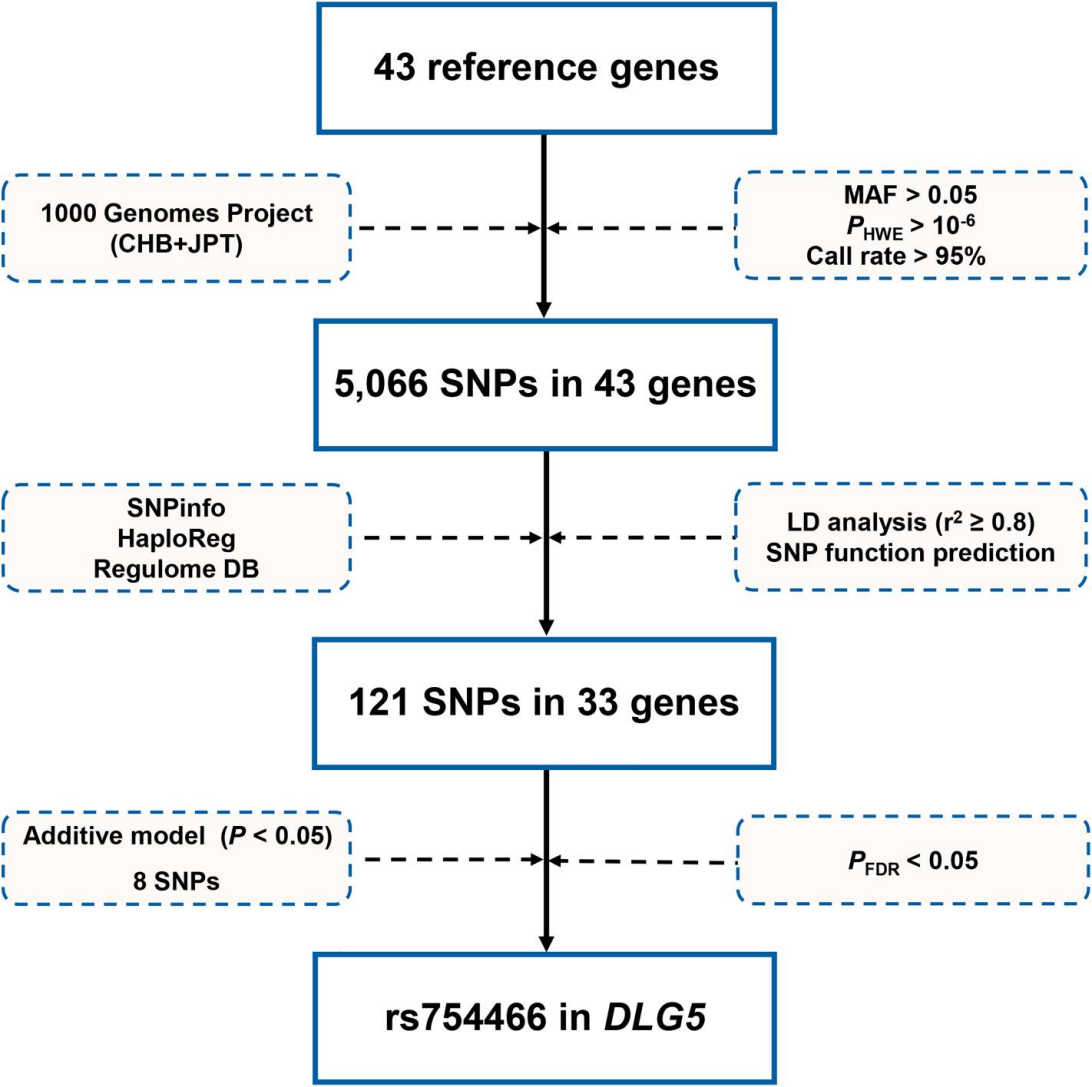
Flow chart for selecting SNPs in the Hippo pathway genes. Forty‐three key genes in the Hippo pathway were selected from the Kyoto Encyclopedia of Genes and Genomes (KEGG), Reactome Pathway database, and reported studies

Of 49 candidate genes from KEGG and Reactome Pathway database, we teased out 43 genes having vital functions in diseases from reported studies (Figure S2 and Table S1). The flow chart of selecting genetic variants is presented in Figure 1. We identified 5066 variants in 43 genes from the 1000 Genomes Project after quality control. Next, 577 genetic variants were filtered out with LD analysis. Then, 121 variants in 33 genes were retained for further study after functional annotation predicted by SNPinfo, HaploReg, and RegulomeDB. In this study, we found that 8 genetic variants (rs754466, rs11002309, rs7744287, rs6790596, rs2032, rs7650899, rs2425672, and rs2236947) were nominally associated with HDM‐induced AR risk in the additive genetic model (all *p* < 0.05) (Table 2). After FDR correction, only rs754466 in *DLG5* was found to be associated with a decreased risk of HDM‐induced AR (*P*_FDR_ = 3.93 × 10^−2^).

**TABLE 2:** Associations of eight significant SNPs with HDM‐induced AR risk

Chr	SNP	Locus	Position^a^	Gene	Allele^b^	EAF^c^	*P*_HWE_	OR (95% CI)	*p*	OR (95% CI)^d^	*p* ^d^	*p*_FDR_
10	rs754466	10q22.3	79,680,434	*DLG5*	T/A	0.12/0.22	2.54 × 10^−1^	0.52 (0.37–0.74)	3.32 × 10^−4^	0.52 (0.36–0.74)	3.25 × 10^−4^	3.93 × 10^−2^
10	rs11002309	10q22.3	79,594,931	*DLG5*	C/T	0.13/0.22	6.31 × 10^−1^	0.55 (0.39–0.79)	1.07 × 10^−3^	0.55 (0.39–0.79)	1.15 × 10^−3^	6.98 × 10^−2^
6	rs7744287	6p21.31	35,463,202	*TEAD3*	G/T	0.37/0.48	4.69 × 10^−3^	0.67 (0.52–0.86)	2.08 × 10^−3^	0.67 (0.52–0.86)	2.11 × 10^−3^	8.50 × 10^−2^
3	rs6790596	3q25.1	149,347,047	*WWTR1*	G/A	0.25/0.32	6.48 × 10^−1^	0.67 (0.50–0.90)	7.35 × 10^–3^	0.67 (0.49–0.90)	7.62 × 10^−3^	2.30 × 10^−1^
15	rs2032	15q13.1	30,112,479	*TJP1*	T/A	0.20/0.14	1.00	1.56 (1.10–2.23)	1.35 × 10^–2^	1.57 (1.10–2.24)	1.26 × 10^−2^	3.04 × 10^−1^
3	rs7650899	3p25.3	11,702,723	*VGLL4*	C/G	0.41/0.50	1.53 × 10^−1^	0.72 (0.56–0.93)	1.29 × 10^–2^	0.72 (0.56–0.93)	1.33 × 10^−2^	2.68 × 10^−1^
20	rs2425672	20q13.12	43,526,137	*YWHAB*	A/G	0.26/0.32	4.18 × 10^−1^	0.72 (0.53–0.98)	3.38 × 10^−2^	0.72 (0.53–0.98)	3.47 × 10^−2^	6.00 × 10^−1^
3	rs2236947	3p21.31	50,371,432	*RASSF1*	C/A	0.09/0.06	3.22 × 10^−1^	1.64 (1.00–2.67)	4.95 × 10^−2^	1.65 (1.01–2.69)	4.73 × 10^−2^	7.15 × 10^−1^

Abbreviations: AR, allergic rhinitis; Chr, chromosome; CI, confidence interval; EAF, effect allele frequency; FDR, false discovery rate; HDM, house dust mite; HWE, Hardy–Weinberg equilibrium; OR, odds ratio; SNP, single nucleotide polymorphism.

^a^ Based on NCBI build 37 of the human genome.

^b^ Reference allele/effect allele.

^c^ Case/control.

^d^ Adjusted for age and sex in the logistic regression model.

To comprehensively investigate the association between rs754466 and HDM‐induced AR risk, we used four genetic models (codominant, additive, dominant, and recessive models) to analyze the correlation of rs754466 with HDM‐induced AR risk (Table 3). The frequencies of TT, TA, and AA were 76.6%, 22.1%, and 1.3% in the cases and 62.4%, 31.6%, and 6.0% in the controls. Compared with the TT genotype, TA and AA genotypes were associated with a decreased risk of HDM‐induced AR (adjusted OR_het_ = 0.57, 95% CI = 0.37–0.87, *p* = 9.16 × 10^−3^; OR_hom_ = 0.18, 95% CI = 0.05–0.65, and *p* = 8.88 × 10^−3^). In the additive model, we observed that individuals with the A allele had a reduced risk of HDM‐induced AR, compared with those with the T allele (adjusted OR = 0.52, 95% CI = 0.36–0.74, *p* = 3.25 × 10^−4^). Compared with the TT genotype, the TA/AA genotypes were associated with a lower risk of HDM‐induced AR (adjusted OR = 0.51, 95% CI = 0.34–0.76, *p* = 1.13 × 10^−3^). Furthermore, the AA genotype was also associated with a decreased risk of HDM‐induced AR compared with TT/TA genotypes (adjusted OR = 0.21, 95% CI = 0.06–0.76, *p* = 1.69 × 10^−2^).

**TABLE 3:** Association between rs754466 in *DLG5* and HDM‐induced AR risk

Genotypes	Cases		Controls		OR (95% CI)	*p*	OR (95% CI)^a^	*p* ^a^
	*N*	%	*N*	%				
TT	170	76.6	148	62.4	1.00		1.00	
TA	49	22.1	75	31.6	0.57 (0.37–0.87)	8.79 × 10^−3^	0.57 (0.37–0.87)	9.16 × 10^−3^
AA	3	1.3	14	6.0	0.19 (0.05–0.66)	9.35 × 10^−3^	0.18 (0.05–0.65)	8.88 × 10^−3^
Additive model					0.52 (0.37–0.74)	3.32 × 10^−4^	0.52 (0.36–0.74)	3.25 × 10^−4^
Dominant model					0.51 (0.34–0.76)	1.13 × 10^−3^	0.51 (0.34–0.76)	1.13 × 10^−3^
Recessive model					0.22 (0.06–0.77)	1.80 × 10^−2^	0.21 (0.06–0.76)	1.69 × 10^−2^

Abbreviations: AR, allergic rhinitis; CI, confidence interval; HDM, house dust mite; OR, odds ratio.

^a^ Adjusted for age and sex in the logistic regression model.

### 3.3 Stratification analyses of rs754466 with HDM‐induced AR risk

**FIGURE 2:**
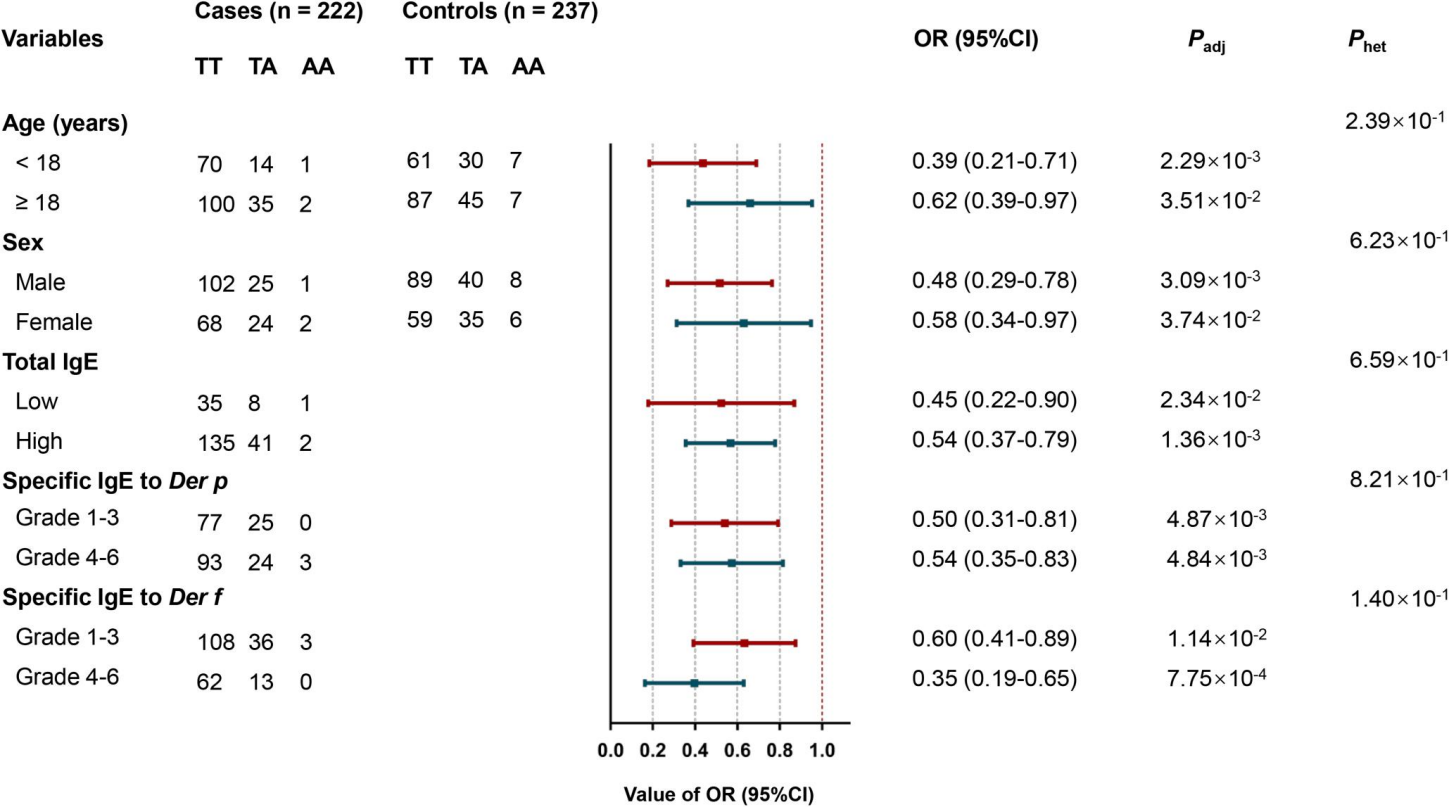
Stratification analyses for the association between rs754466 and HDM‐induced AR risk in the additive model. A logistic regression model with adjustments for age and sex was constructed to calculate adjusted odds ratios (ORs), and their 95% confidence intervals (CIs). *P*_adj_, *p* value adjusted for age and sex; *P*_het_, *p* value for heterogeneity

Subgroup analyses based on different demographic characteristics were performed (Figure 2 and Table S2). The rs754466 A allele was associated with a lower risk in age and sex subgroups (all *p* < 0.05). Additionally, the rs754466 A allele decreased the risk of HDM‐induced AR in all subgroups of total IgE, specific IgE to *Der p*, and specific IgE to *Der f* (all *p* < 0.05). However, no significant heterogeneity was observed in all these subgroup analyses (all *p* > 0.05).

**FIGURE 3:**
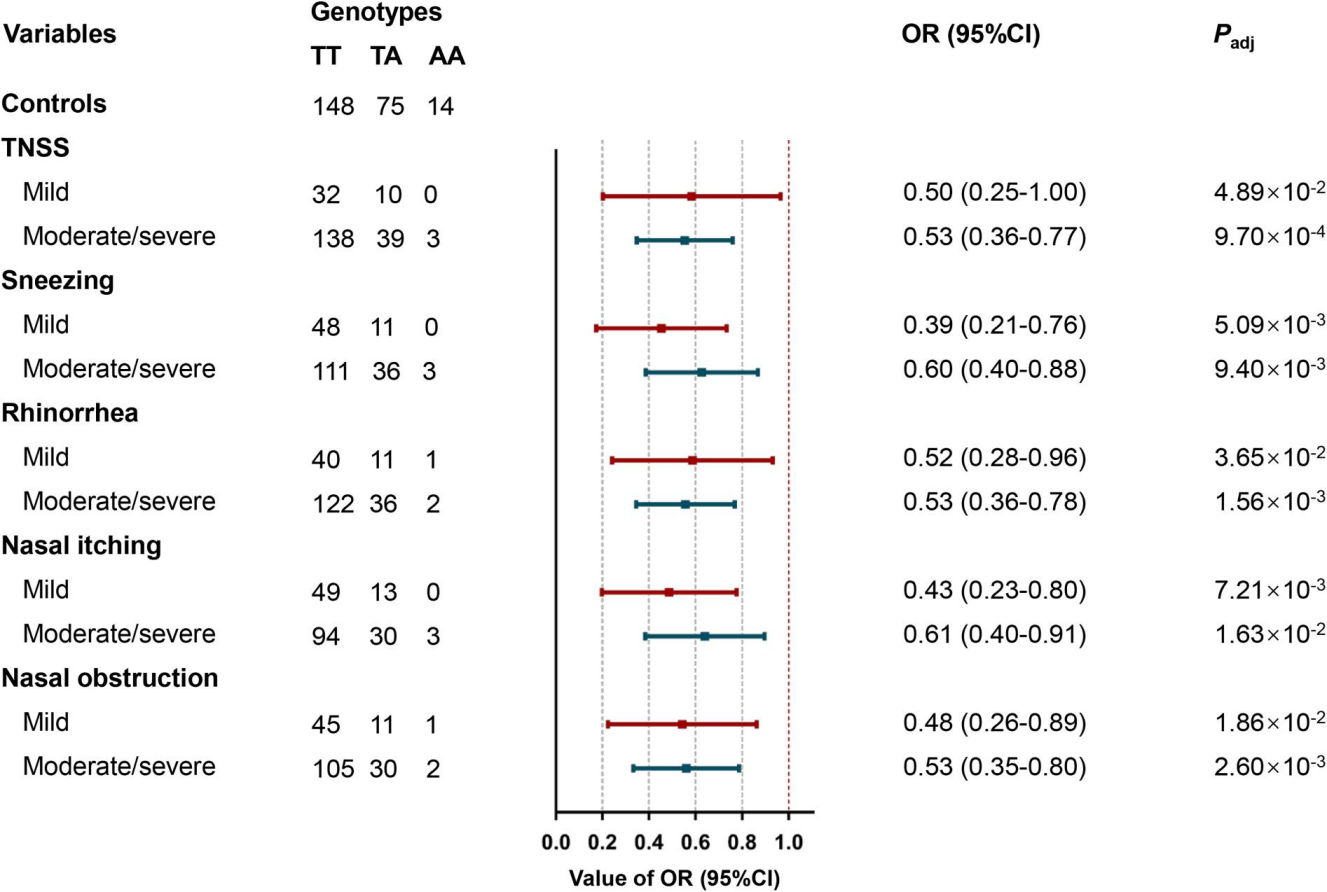
Stratification analyses of clinical features for the association between rs754466 and HDM‐induced AR risk in the additive model. A logistic regression model with adjustments for age and sex was constructed to calculate adjusted odds ratios (ORs), and their 95% confidence intervals (CIs). *P*_adj_, *p* value adjusted for age and sex

In the moderate/severe TNSS subgroup, the rs754466 A allele showed the most significant association with a reduced risk of HDM‐induced AR (*p* = 9.70 × 10^−4^). A stronger association was also observed between the rs754466 A allele and a decreased HDM‐induced AR risk in moderate/severe rhinorrhea, moderate/severe nasal obstruction, mild sneezing and mild nasal itching subgroups (Figure 3 and Table S3).

### 3.4 Function of rs754466 in *DLG5*

Using SNPinfo, HaploReg and RegulomeDB, we observed that rs754466 possessed enhancer histone marks, DNAse hypersensitivity sites, altered motifs, GRASP hit, and selected eQTL hits (Table S4). Moreover, we found that histone modification and transcription factor binding sites were rich in the region harboring rs754466 by UCSC genome browser (Figure S3). In addition, the rs754466 T allele was associated with a high expression of *DLG5* in whole blood, lung and cultured fibroblasts based on GTEx database. However, rs754466 mutated in a manner of A > T in the public database, but T > A in our study. This inconsistency may be explained by that the DNA strand detected in our study was complementary to that detected in public database. Moreover, similar results were reported by Wang et al.^24^ and Feng et al.^25^ Hence, the rs754466 A allele in our study increased the expression of *DLG5* in whole blood, lung, and cultured fibroblasts (Figure S4).

## 4 DISCUSSION

In mammals, the Hippo pathway is mainly composed of mammalian STE20‐like protein kinase half (MST1/2), Salvador family WW domain‐containing protein 1 (SAV1), large tumor suppressor half (LATS1/2), MOB kinase activator 1 A/B (MOB1A/B), Yes‐associated protein (YAP), and transcriptional co‐activator with PDZ‐binding motif (TAZ).^22^, ^23^ The Hippo pathway regulates the proliferation of airway epithelial cells.^26^, ^27^, ^28^, ^29^ When the Hippo pathway is suppressed, YAP and TAZ translocate to the nucleus to induce expression of target genes, thus causing asthma, lung fibrosis and chronic rhinosinusitis with nasal polyps (CRSwNP).^12^, ^13^, ^14^, ^22^ It was reported that the susceptibility to HDM‐induced AR was closely related to genetic variations.^30^ lesions,Previous study indicated that rs9671722 in the Hippo pathway increased the risk of AR with asthma.^13^ To our knowledge, no study has reported the association between genetic variants in the Hippo pathway genes and HDM‐induced AR risk. In the present study, we found that rs754466 in *DLG5* was strongly correlated with a decreased risk of HDM‐induced AR.

*DLG5*, discs large MAGUK scaffold protein 5, encodes a membrane‐associated guanylate kinase (MAGUK) containing a coiled‐coil domain, a GUK domain, a SH3 domain, a CARD domain, a Duff domain, and four PDZ domains.^31^ Recent studies have reported that *DLG5* can increase the phosphorylation of YAP and TAZ to inhibit the nuclear translocation of YAP and TAZ.^19^, ^32^ TAZ promotes Th17 cells differentiation and attenuates regulatory T cells development,^33^ which might serve as a mechanism in the pathogenesis of AR.^34^ Additionally, DLG5 maintains the integrity of epithelial barrier to curb the invasion of pathogens into the tissue interstitium.^35^, ^36^ As previously described, *DLG5* lowly expressed in Crohn's disease,^35^ breast cancer,^32^ prostate cancer,^37^ and hepatocellular carcinoma.^38^ Moreover, previous studies have reported that *DLG5* downregulation leads to emphysema‐like lesions,^31^ indicating that *DLG5* might function in respiratory diseases. Notably, HDM may initiate HDM‐induced AR through impairing nasal epithelial barrier.^39^ For the first time, therefore, we speculated that *DLG5* might participate in the pathogenesis of HDM‐induced AR. However, the expression of *DLG5* is required to be detected for further validating our hypothesis. And further biological function experiments are necessary to fully illuminate the detailed molecular mechanisms underlying the role of *DLG5* in HDM‐induced AR.

Many genetic variants in *DLG5* are significantly associated with Crohn's disease susceptibility.^40^ However, the associations between genetic variants in *DLG5* and HDM‐induced AR have not been reported. In the stratification analyses, we found that the rs754466 A allele decreased the risk of HDM‐induced AR in all subgroups of age and sex, indicating that this variant might be of predictive value for HDM‐induced AR population. Furthermore, rs754466 in *DLG5* had a significantly reduced risk of HDM‐induced AR in subgroups of serum indexes (total IgE and specific IgEs) and nasal symptoms. The nasal epithelial barrier dysfunction results in an increased allergen passage and then B cells produce IgE after allergens uptake.^41^, ^42^ Moreover, the severity of nasal symptoms is correlated with status of nasal barrier.^43^ Notably, DLG5 maintains the integrity of epithelial barrier, preventing pathogens from invading into the tissue interstitium.^35^, ^36^ These results suggested that rs754466 might strengthen the epithelial barrier function by regulating the expression of *DLG5*, thereby restraining the allergens intrusion and thus decreasing IgE production and the occurrence of nasal symptoms. This hypothesis may be supported by our findings in GTEx database. Although nasal tissues were unavailable in the GTEx database, we observed that the rs754466 A allele came with a high expression of *DLG5* in lung tissues. Given the anatomic relationship between nose and lung (located at the upper and lower respiratory tract, respectively), we dared to speculate that the rs754466 A allele might increase the expression of *DLG5* in nasal tissues. As a consequence, the nuclear translocation of TAZ was inhibited and barrier function was strengthened, all contributing to the lower risk of HDM‐induced AR. These findings may refresh our understanding of the pathogenesis, provide a novel target for early prevention and precision treatment of HDM‐induced AR.

Our study has some limitations. First of all, AR is a complex disease induced by gene‐environment interactions. Many environmental factors such as nitric oxides, sulfur pollutants, organic chemical agents, particulate matter and tobacco smoke have been shown associated with AR risk.^1^ Thus, environmental factors in the study population should be investigated. Second, neither next‐generation sequencing nor genome‐wide approach was used in our study. Therefore, these genetic techniques in large HDM‐induced AR cases and controls are needed to systematically validate our findings and reveal novel susceptibility loci of HDM‐induced AR. Third, the expression of DLG5 in nasal tissues should be analyzed. Finally, the study may benefit from experiments of SNP rs7454466 and the functionally active proteins related to the SNP. Future experiments including cell transfection, Western blotting, quantitative real‐time PCR, chromatin immunoprecipitation assay and electrophoretic mobility shift assay should be conducted to test the function of SNP and these proteins.

## 5 CONCLUSIONS

We performed the first analysis for associations between genetic variations in the Hippo pathway genes and the risk of HDM‐induced AR in a Chinese population. And we found that rs754466 in *DLG5* had a significant decreased risk of HDM‐induced AR. Our findings might provide new insight into the pathogenesis of HDM‐induced AR and a new therapeutic target.

## CONSENT FOR PUBLICATION

All participants signed their written consent for publication.

## CONFLICT OF INTEREST

The authors declare that they have no competing interests.

## Supporting information

Supporting Information S1
