# Supplementary material for: Genetic variants in Hippo pathway genes are associated with house dust mite‐induced allergic rhinitis in a Chinese population
Source: Clin Transl Allergy. 2021 Dec 28;11(10):e12077. doi: 10.1002/clt2.12077 (PMC8805694; doi:10.1002/clt2.12077)

**Table S1. The key genes of the Hippo pathway.**

| **Chr** | **Gene** | **Position^a^** |
| --- | --- | --- |
| 1 | *NPHP4* | 5922870-6052618 |
| 1 | *PTPN14* | 214521640-214725024 |
| 1 | *TP53BP2* | 223967595-224033674 |
| 2 | *MOB1A* | 74381394-74406006 |
| 3 | *AMOTL2* | 134074187-134094321 |
| 3 | *LIMD1* | 45636323-45722755 |
| 3 | *RASSF1* | 50367217-50378367 |
| 3 | *VGLL4* | 11597543-11762220 |
| 3 | *WWTR1* | 149235022-149421060 |
| 3 | *DLG1* | 196769431-197026171 |
| 4 | *ANKRD17* | 73940502-74124858 |
| 4 | *MOB1B* | 71768043-71853891 |
| 4 | *RASSF6* | 74437267-74486348 |
| 5 | *ANKHD1* | 139781399-139919441 |
| 5 | *WWC1* | 167719065-167899308 |
| 6 | *LATS1* | 149979289-150039392 |
| 6 | *TEAD3* | 35441374-35464884 |
| 8 | *STK3* | 99466859-99954799 |
| 8 | *SCRIB* | 144873090-144897890 |
| 9 | *TJP2* | 71714875-71870124 |
| 10 | *BTRC* | 103113790-103317078 |
| 10 | *DLG5* | 79550549-79686348 |
| 11 | *AMOTL1* | 94439602-94609918 |
| 11 | *PPP1CA* | 67165652-67169376 |
| 11 | *YAP1* | 101981151-102104154 |
| 11 | *TEAD1* | 12695969-12966284 |
| 11 | *DLG2* | 83166055-85338314 |
| 12 | *TEAD4* | 3068478-3149842 |
| 13 | *LATS2* | 21547175-21635722 |
| 14 | *AJUBA* | 23440386-23451851 |
| 14 | *FRMD6* | 51955819-52197445 |
| 14 | *SAV1* | 51100298-51135071 |
| 15 | *TJP1* | 29992338-30261308 |
| 17 | *DVL2* | 7128661-7137867 |
| 17 | *TAOK1* | 27717943-27878921 |
| 17 | *YWHAE* | 1247833-1303669 |
| 17 | *LLGL1* | 18128907-18148189 |
| 17 | *BIRC5* | 76210277-76221716 |
| 19 | *WTIP* | 34972880-34992085 |
| 19 | *TEAD2* | 49843852-49865714 |
| 20 | *STK4* | 43595120-43708600 |
| 20 | *YWHAB* | 43514240-43537173 |
| 22 | *NF2* | 29999545-30094589 |

^a^Based on NCBI build 37 of the human genome.

**Table S2. Stratification analyses for the association between rs754466 and HDM-induced AR risk in additive model.**

| **Variables** | **Cases (n = 222)** | | |  | **Controls (n = 237)** | | | **OR (95%CI)^a^** | ***P*^a^** | ***P*^b^** |
| --- | --- | --- | --- | --- | --- | --- | --- | --- | --- | --- |
|  | **TT, N (%)** | **TA, N (%)** | **AA, N (%)** |  | **TT, N (%)** | **TA, N (%)** | **AA, N (%)** |  |  |  |
| Age |  |  |  |  |  |  |  |  |  | 2.39×10^-1^ |
| < 18 | 70 (82.3) | 14 (16.5) | 1 (1.2) |  | 61 (62.2) | 30 (30.6) | 7 (7.2) | 0.39 (0.21-0.71) | 2.29×10^-3^ |  |
| ≥ 18 | 100 (73.0) | 35 (25.5) | 2 (1.5) |  | 87 (62.6) | 45 (32.4) | 7 (5.0) | 0.62 (0.39-0.97) | 3.51×10^-2^ |  |
| Sex |  |  |  |  |  |  |  |  |  | 6.23×10^-1^ |
| Male | 102 (79.7) | 25 (19.5) | 1 (0.8) |  | 89 (65.0) | 40 (29.2) | 8 (5.8) | 0.48 (0.29-0.78) | 3.09×10^-3^ |  |
| Female | 68 (72.3) | 24 (25.5) | 2 (2.2) |  | 59 (59.0) | 35 (35.0) | 6 (6.0) | 0.58 (0.34-0.97) | 3.74×10^-2^ |  |
| Total IgE |  |  |  |  |  |  |  |  |  | 6.59×10^-1^ |
| Low^c^ | 35 (79.5) | 8 (18.2) | 1 (2.3) |  |  |  |  | 0.45 (0.22-0.90) | 2.34×10^-2^ |  |
| High^d^ | 135 (75.8) | 41 (23.0) | 2 (1.2) |  |  |  |  | 0.54 (0.37-0.79) | 1.36×10^-3^ |  |
| Specific IgE to *Der p* |  |  |  |  |  |  |  |  |  | 8.21×10^-1^ |
| Grade 1-3 | 77 (75.5) | 25 (24.5) | 0 (0.0) |  |  |  |  | 0.50 (0.31-0.81) | 4.87×10^-3^ |  |
| Grade 4-6 | 93 (77.5) | 24 (20.0) | 3 (2.5) |  |  |  |  | 0.54 (0.35-0.83) | 4.84×10^-3^ |  |
| Specific IgE to *Der f* |  |  |  |  |  |  |  |  |  | 1.40×10^-1^ |
| Grade 1-3 | 108 (73.5) | 36 (24.5) | 3 (2.0) |  |  |  |  | 0.60 (0.41-0.89) | 1.14×10^-2^ |  |
| Grade 4-6 | 62 (82.7) | 13 (17.3) | 0 (0.0) |  |  |  |  | 0.35 (0.19-0.65) | 7.75×10^-4^ |  |

HDM, house dust mite; AR, allergic rhinitis; OR, odds ratio; CI, confidence interval; *Der p*, *Dermatophagoides pteronyssinus*; *Der f*, *Dermatophagoides farinae*.

^a^Adjusted for age and sex in logistic regression model.

^b^*P* value for heterogeneity.

^c^Total IgE < 60.45 kU/L.

^d^Total IgE ≥ 60.45 kU/L.

**Table S3. Stratification analyses of clinic features for the association between rs754466 and HDM-induced AR risk in additive model.**

| **Variables** | **Genotype** | | |  | **OR (95%CI)^a^** | ***P*^a^** |
| --- | --- | --- | --- | --- | --- | --- |
|  | **TT, N (%)** | **TA, N (%)** | **AA, N (%)** |  |  |  |
| Controls | 148 (62.4) | 75 (31.6) | 14 (6.0) |  |  |  |
| TNSS |  |  |  |  |  |  |
| Mild^b^ | 32 (76.2) | 10 (23.8) | 0 (0.0) |  | 0.50 (0.25-1.00) | 4.89×10^-2^ |
| Moderate/severe^c^ | 138 (76.7) | 39 (21.7) | 3 (1.6) |  | 0.53 (0.36-0.77) | 9.70×10^-4^ |
| Symptom: sneezing |  |  |  |  |  |  |
| Mild | 48 (81.4) | 11 (18.6) | 0 (0.0) |  | 0.39 (0.21-0.76) | 5.09×10^-3^ |
| Moderate/severe | 111 (74.0) | 36 (24.0) | 3 (2.0) |  | 0.60 (0.40-0.88) | 9.40×10^-3^ |
| Symptom: rhinorrhea |  |  |  |  |  |  |
| Mild | 40 (77.0) | 11 (21.1) | 1 (1.9) |  | 0.52 (0.28-0.96) | 3.65×10^-2^ |
| Moderate/severe | 122 (76.3) | 36 (22.5) | 2 (1.2) |  | 0.53 (0.36-0.78) | 1.56×10^-3^ |
| Symptom: nasal itching |  |  |  |  |  |  |
| Mild | 49 (79.0) | 13 (21.0) | 0 (0.0) |  | 0.43 (0.23-0.80) | 7.21×10^-3^ |
| Moderate/severe | 94 (74.0) | 30 (23.6) | 3 (2.4) |  | 0.61 (0.40-0.91) | 1.63×10^-2^ |
| Symptom: nasal obstruction |  |  |  |  |  |  |
| Mild | 45 (78.9) | 11 (19.3) | 1 (1.8) |  | 0.48 (0.26-0.89) | 1.86×10^-2^ |
| Moderate/severe | 105 (76.6) | 30 (21.9) | 2 (1.5) |  | 0.53 (0.35-0.80) | 2.60×10^-3^ |

HDM, house dust mite; AR, allergic rhinitis; OR, odds ratio; CI, confidence interval; TNSS, total nasal symptom score.

^a^Adjusted for age and sex in logistic regression model.

^b^TNSS ≤ 4.

^c^TNSS > 4.

**Table S4. Functional annotation of 8 SNPs in silico analysis.**

| **Chr** | **SNP** | **Locus** | **Position^a^** | **Gene** | **Allele^b^** | **MAF^c^** | **Score^d^** | **Promoter**  **histone marks** | **Enhancer**  **histone marks** | **DNAse** | **Proteins bound** | **Motifs changed** | **GRASP**  **QTL hits** | **Selected**  **eQTL hits** | **Total**  **score** |
| --- | --- | --- | --- | --- | --- | --- | --- | --- | --- | --- | --- | --- | --- | --- | --- |
| 3 | rs2236947 | 3p21.31 | 50371432 | *RASSF1* | C/A | 0.13 | 1f | 5 tissues | 18 tissues | 23 tissues |  | Pax-6 | 13 hits | 50 hits |  |
| 20 | rs2425672 | 20q13.12 | 43526137 | *YWHAB* | A/G | 0.32 | 1f | BLD | 10 tissues | IPSC,BLD |  |  | 2 hits | 16 hits |  |
| 6 | rs7744287 | 6p21.31 | 35463202 | *TEAD3* | G/T | 0.38 | 2b | 17 tissues | 16 tissues | 19 tissues | RFX5 | 10 altered motifs |  | 1 hit |  |
| 10 | rs11002309 | 10q22.3 | 79594931 | *DLG5* | C/T | 0.23 | 2b |  | 9 tissues | MUS |  | 6 altered motifs | 1 hit | 50 hits |  |
| 10 | rs754466 | 10q22.3 | 79680434 | *DLG5* | A/T | 0.22 | 3a |  | 15 tissues | 4 tissues |  | 7 altered motifs | 1 hit | 25 hits |  |
| 3 | rs7650899 | 3p25.3 | 11702723 | *VGLL4* | G/C | 0.47 | 3a | LNG | 14 tissues | 8 tissues | CFOS |  |  |  |  |
| 3 | rs6790596 | 3q25.1 | 149347047 | *WWTR1* | G/A | 0.38 | 4 |  | 12 tissues | ESDR,OVRY | FOXA1 | AP-1,Pbx-1 |  | 2 hits |  |
| 15 | rs2032 | 15q13.1 | 30112479 | *TJP1* | A/T | 0.13 | 4 | 21 tissues | 4 tissues | 9 tissues |  | 14 altered motifs |  |  |  |

SNP, single nucleotide polymorphism.

^a^Based on NCBI build 37 of the human genome.

^b^Major allele/minor allele.

^c^Minor allele frequency in 1000 Genomes Project East Asian data.

^d^Based on RegulomeDB.

**Supplementary Figure legends**

**Fig S1. Receiver operating characteristic curve of serum total IgE levels.** The cut-off value of total IgE for the diagnosis of individuals with HDM-induced AR was set as 60.45 kU/L.

**Fig S2. Association between Hippo pathway and respiratory diseases.** The yellow round rectangles indicate the key components of the Hippo pathway.

**Fig S3. *In* *silico* analysis for rs754466 functional annotation.** The histone modification and transcription factor binding sites were from ChIP-Seq data deposited in ENCODE and visualized by UCSC genome browser.

**Fig S4. The eQTL analysis in GTEx database.** The effect of rs754466 on the expression of *DLG5* in GTEx database in whole blood (a), lung (b), and cultured fibroblasts (c).

**Fig S1.**


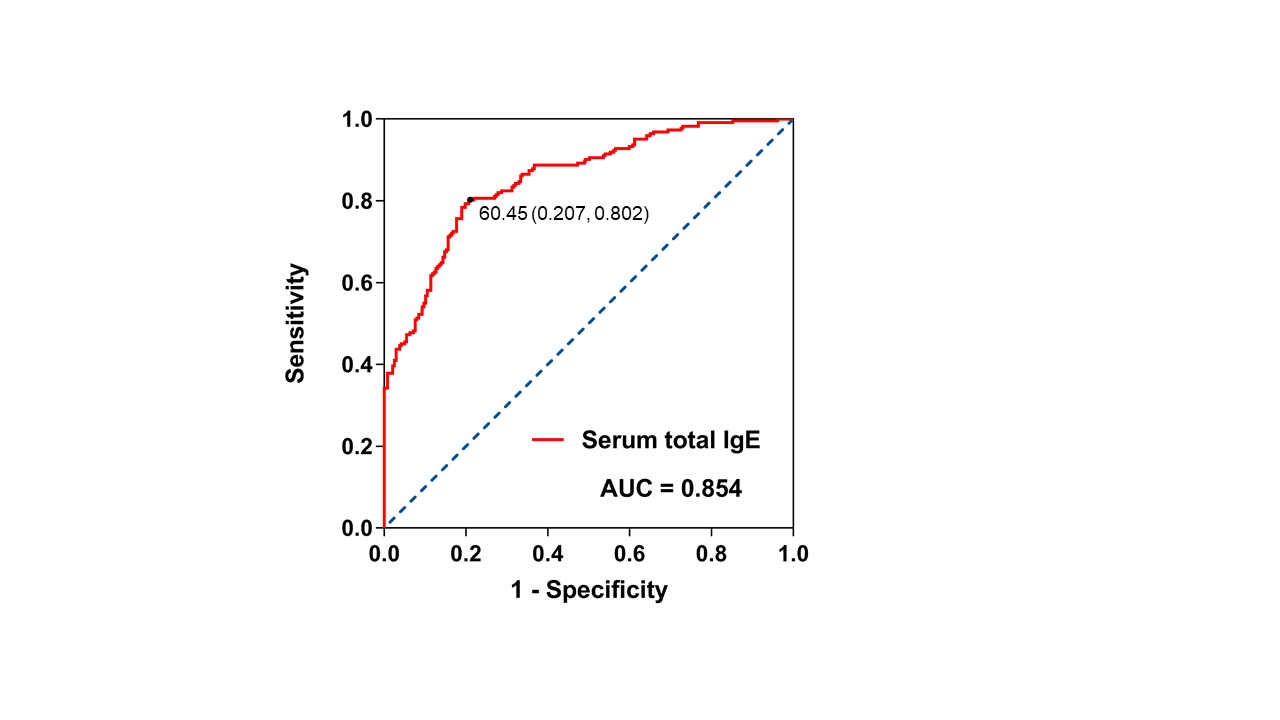


**Fig S2.**


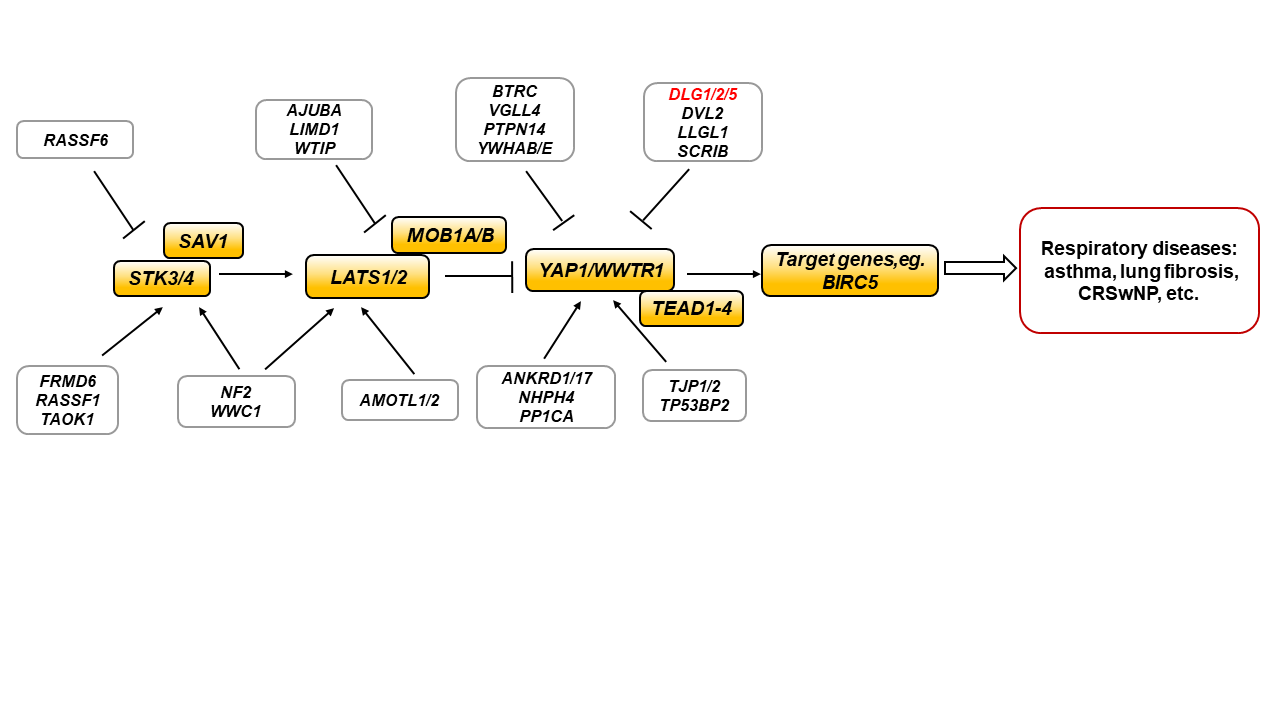


**Fig S3.**


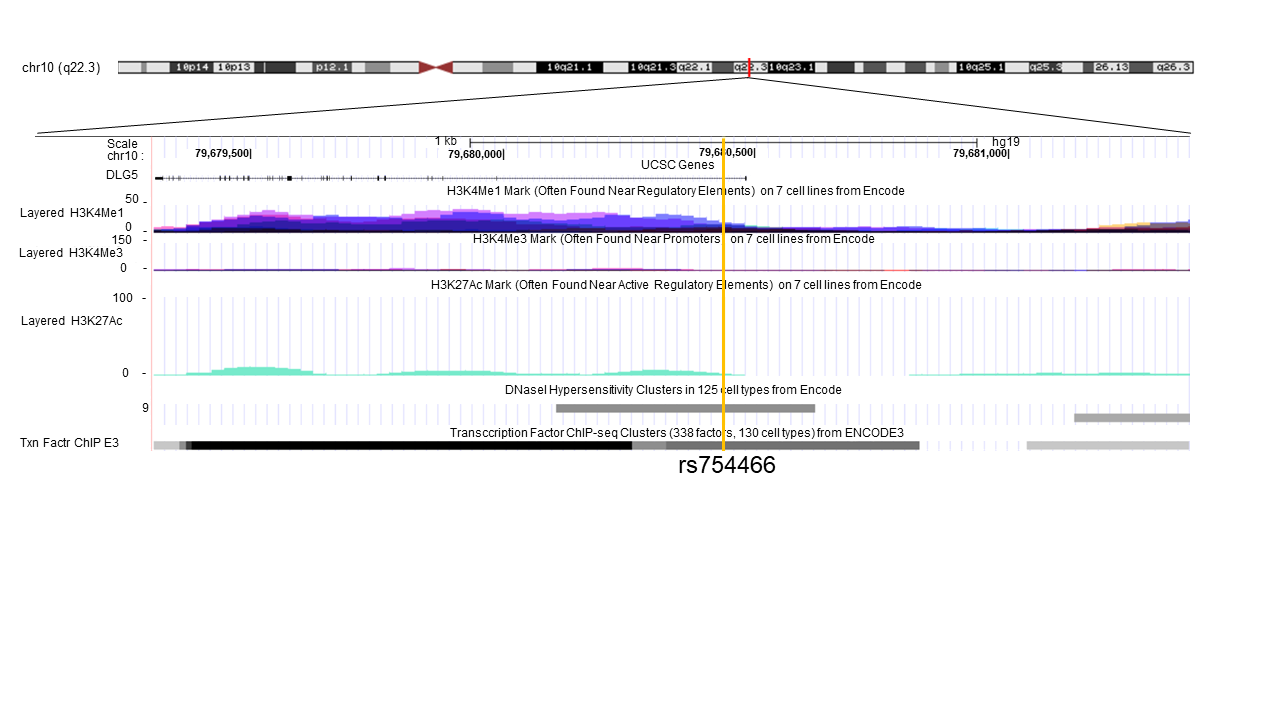


**Fig S4.**


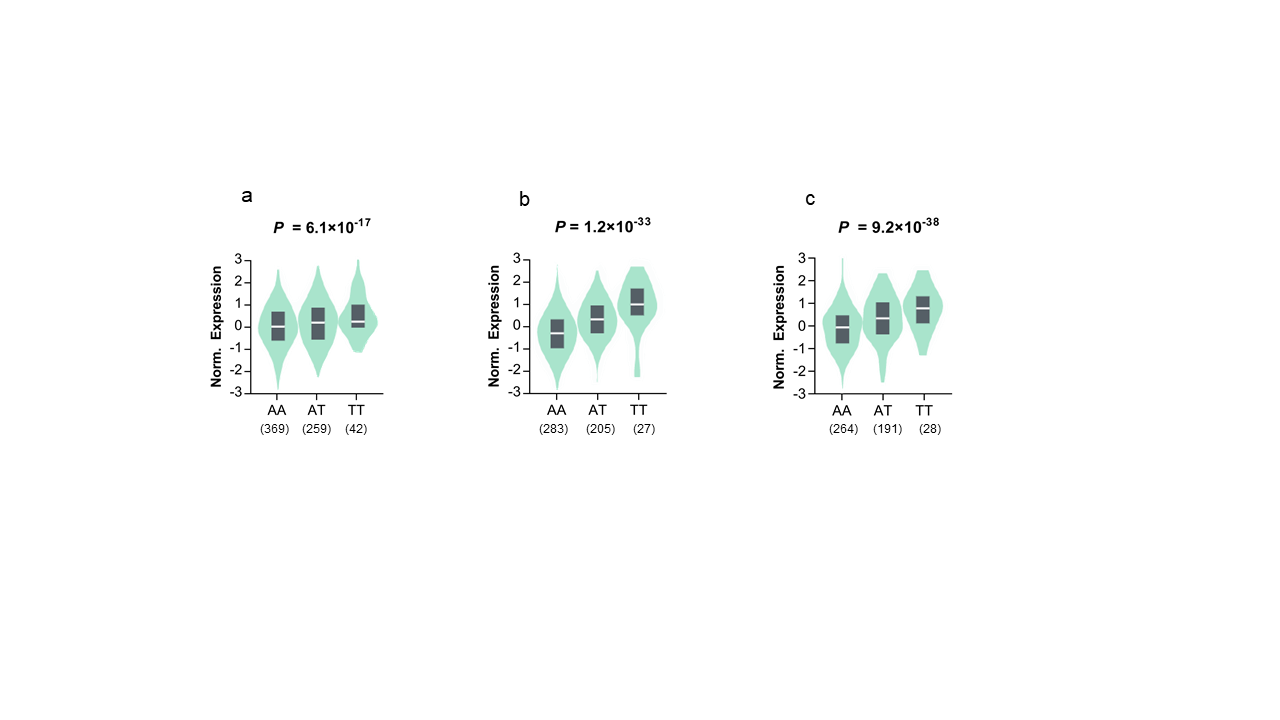

Supplement: Supplementary file 1 — Supporting Information S1 [file CLT2-11-e12077-s001.docx]
